# Perceived Self‐Efficacy and Its Determinants for Noncommunicable Disease Prevention Among Adults in Southern Ethiopia: A Community‐Based Cross‐Sectional Study

**DOI:** 10.1155/bmri/3904968

**Published:** 2026-07-01

**Authors:** Habtamu Endashaw Hareru, Mesfin Abebe, Berhanu G. Debela, Tizalegn Tesfaye Mamo, Yohannes Addisu Wondimagegne

**Affiliations:** ^1^ School of Public Health, College of Health Sciences and Medicine, Dilla University, Dilla, Ethiopia, du.edu.et; ^2^ Wonago Health and Demographic Surveillance System, Dilla University, Wonago, Ethiopia, du.edu.et; ^3^ Department of Midwifery, College of Health Sciences and Medicine, Dilla University, Dilla, Ethiopia, du.edu.et

**Keywords:** disease prevention, health belief model, noncommunicable diseases, perceived self-efficacy, Southern Ethiopia

## Abstract

**Background:**

Noncommunicable diseases (NCDs) are the leading causes of morbidity and mortality worldwide. The burden of NCD‐related morbidity and mortality can be reduced through the adoption of healthy lifestyle behaviors and the utilization of preventive healthcare services. Self‐efficacy plays a crucial role in maintaining healthy behavior and improving health outcomes. However, there is limited evidence regarding the magnitude of perceived self‐efficacy in the study area. Therefore, this study is aimed at assessing the magnitude of perceived self‐efficacy and its associated factors in preventing NCDs among adult residents in the Gedeo Zone, Southern Ethiopia.

**Methods:**

A community‐based cross‐sectional study was conducted from November 1, 2023, to January 1, 2024, among adult residents of the Gedeo Zone, Southern Ethiopia. A multistage sampling technique was employed to select 620 participants. Data were collected using interview‐administered structured questionnaires through KoboToolbox and exported to STATA Version 16 for analysis. Descriptive statistics were computed, and binary logistic regression analysis was performed to identify candidate variables associated with perceived self‐efficacy in preventing NCDs. Variables with *p* value < 0.05 in the multivariable model were considered statistically significant. Adjusted odds ratios (AORs) with 95% confidence intervals (CIs) were calculated and reported.

**Results:**

Overall, 56.5% (95% CI: 52.5%–60.3%) of participants had high perceived self‐efficacy toward engaging in NCD preventive behaviors. Middle‐aged adults (AOR = 1.81; 95% CI: 1.09, 3.01), older adults (AOR = 4.31; 95% CI: 2.08, 8.07), participants reporting good perceived general health status (AOR = 2.94; 95% CI: 1.38, 6.28), perceived benefit of health action (AOR = 5.54; 95% CI: 3.32, 9.25), and perceived cues to action (AOR = 4.35; 95% CI: 2.75, 6.88) were positively associated with high perceived self‐efficacy in preventing NCDs. However, perceived barriers to health actions (AOR = 0.58; 95% CI: 0.38, 0.88) were negatively associated with high perceived self‐efficacy.

**Conclusion and Recommendations:**

The study found that more than half of the participants demonstrated high perceived self‐efficacy in preventing NCDs. Being middle or older, having a good perceived general health status, perceiving benefits of health actions, and having cues to action were positively associated with self‐efficacy, whereas perceived barriers to health actions were negatively associated with perceived self‐efficacy. These findings suggest that policymakers and healthcare providers should strengthen community‐based health promotion and social and behavior change interventions aimed at improving individuals′ efficacy in engaging in NCD preventive behaviors.

## 1. Introduction

Noncommunicable diseases (NCDs) are long‐term conditions characterized by multifactorial causation, involving both nonmodifiable determinants such as genetic predisposition and modifiable risk factors, including metabolic, behavioral, environmental, and social influences NCDs [1–3]. The principal categories of NCDs comprise cardiovascular diseases (e.g., heart attack and stroke), cancer, diabetes, and chronic respiratory diseases (i.e., asthma and chronic obstructive pulmonary disease) [4]. These four major NCD categories share common modifiable behavioral risk factors, including unhealthy diet, tobacco use, harmful alcohol consumption, and physical inactivity. In addition, environmental exposures, particularly air pollution, significantly contribute to the global burden of NCDs. NCDs represent a major global health challenge, placing substantial pressure on health systems and economic development, particularly in low‐ and middle‐income countries [5]. Reducing these risk factors across the population is important in reducing rates of premature mortality and the global burden of NCDs. People living with or at risk of NCDs should therefore be encouraged to adopt healthy behaviors to reduce disease risk and improve overall health outcomes [6–8].

In 2021, NCDs were responsible for an estimated 43 million deaths worldwide, accounting for approximately 75% of all nonpandemic‐related global mortality. Of these deaths, nearly 18 million occurred prematurely among individuals under the age of 70 years, with more than 80% of such premature deaths taking place in low‐ and middle‐income countries. Overall, these countries bear a disproportionate burden, accounting for nearly three‐quarters of total NCD‐related mortality [1].

According to the United Nations (UN) sustainable development agenda, the goal is to reduce premature mortality (between the ages of 30 and 70 years) from the four main NCDs (cardiovascular disease, cancer, diabetes, and chronic respiratory diseases) by one‐third by 2030 relative to 2015 levels, and to promote mental health and well‐being [9]. To achieve this, it is crucial that countries implement cost‐effective, evidence‐based, feasible, and affordable interventions at both the population and individual levels [10]. Ethiopia is among the sub‐Saharan African countries undergoing epidemiological, nutritional, and demographic changes contributing to the increase in NCDs [11, 12]. In a systematic review and meta‐analysis study, one‐third of Ethiopians had an NCD diagnosis [13].

The health belief model (HBM) is considered the most effective framework for analyzing and predicting health‐related behaviors [14]. This model suggests that individuals are more likely to take action to NCDs if they perceive themselves as at risk (perceived susceptibility), believe that NCDs can have serious consequences (perceived severity), recognize that adopting healthy behaviors may reduce their risk or the severity of the diseases (perceived benefits), identify obstacles to changing their behaviors (perceived barriers), and receive motivation or support to take action (cues to action) [15]. Additionally, this study focuses on another important aspect of the model: self‐efficacy. This concept indicates that individuals are more likely to engage in preventive behaviors if they believe they have the ability to do so. Essentially, self‐efficacy reflects a person′s confidence in their capacity to perform healthy behaviors regarding NCDs [15, 16].

Self‐efficacy is essential for lowering the prevalence of NCDs through resisting perceived barriers to take preventive action and enhancing health outcomes. A key component of managing and preventing NCDs is the perception of self‐efficacy, which is crucial for complex tasks and long‐term modifications in health behavior [17]. Additionally, it serves as a predictor of significant health outcomes like hospitalizations and health‐related quality of life [17, 18], and clinical research has shown that higher self‐efficacy leads to more self‐management practices [19].

Increased likelihood of participating in lifestyle activities that promote health, such as physical activity and a balanced diet, is correlated with higher self‐efficacy [20]. It may also serve as a link between successful health promotion, educational initiatives, and changes in health behaviors in disease control [21]. Therefore, for healthcare providers to create focused interventions that boost people′s confidence in their capacity to prevent NCDs, they need to understand the role that self‐efficacy plays in society at large and communities in particular settings. Despite the growing burden of NCDs in Ethiopia, preventive health behaviors remain limited. Understanding psychosocial determinants such as perceived self‐efficacy is essential for designing effective health promotion interventions. However, evidence regarding perceived self‐efficacy and its associated factors in the prevention of NCDs remains scarce, particularly in southern Ethiopia. Therefore, this study is aimed at assessing the level of perceived self‐efficacy and its associated factors in preventing NCDs among adult residents of the Gedeo Zone, Southern Ethiopia. The findings of the study may help to advance the field of NCD prevention and health promotion by offering important insight and evidence for effective strategies, interventions, or methods that can be used to deal with these diseases. Moreover, the public health policies, guidelines, and programs aimed at promoting healthy behavior and preventing NCDs may benefit from the study′s findings.

## 2. Materials and Methods

This study′s area description, source population, and other methodologies were adopted from a previously published article titled “Health‐Promoting Behavior and Its Determinants Towards Non‐Communicable Diseases Among Adult Residents of the Gedeo Zone, South Ethiopia: The Application of the Health Belief Model [22].” Both studies are part of a research project titled “Community Risk Perception and Health Promotion Behavior Towards Common NCDs Among Adult Residents of the Gedeo Zone, Southern Ethiopia,” which was funded by Dilla University in 2022.

### 2.1. Study Area, Design, and Period

This study was conducted among residents of the Gedeo Zone, located in the South Ethiopia region, which is the newly formed 12th regional state in Ethiopia. The Gedeo Zone extends south as a narrow strip of land along the eastern escarpment of the Ethiopian Highlands and borders the Oromia region to the east, south, and west. To the north, it shares a boundary with the Sidama region. The zone comprises eight woredas: Bule, Gedeb, Wonago, Kochere, Dilla Zuriya, Chorso, Reppe, and Yirgachefe, as well as four administrative towns: Dilla, Gedeb, Chelelektu, and Yirgachefe. In 2014, there were a total of 250,363 households in the zone (source: Gedeo Zone Health Office). Dilla, the zonal town of Gedeo, is situated 359 km south of the Ethiopian capital, Addis Ababa. A community‐based cross‐sectional study was conducted to assess perceived self‐efficacy and factors associated with the prevention of NCDs among adult residents of the Gedeo Zone from November 1, 2023, to January 1, 2024.

### 2.2. Study Population

The target population was all adults aged 18 years or older residing in the Gedeo Zone. The source population consisted of adults aged 18 years or older living in the selected districts (woredas) of the Gedeo Zone during the study period. The study population included adults aged 18 years or older who were permanently residing in the selected households and met the inclusion criteria during the data collection period. Individuals aged 18 years or older who had been residing in the study area for more than 6 months were included in the study. Individuals with previously diagnosed NCDs were excluded from the study to focus on preventive behaviors among apparently healthy adults and those with cognitive impairments that affected their ability to respond were excluded from the study.

### 2.3. Sample Size, Sampling Techniques, and Procedures

To include the eligible participants, the sample size was determined using a single population formula: *n* = (*Z*(*a*/ 2))^2^ *p*(1 − *p*)/*d* ^2^ ; where *n* is the sample size, *d* is the 5% margin of error, Z_*α*/2_ is the two‐sided confidence level (= 1.96 on the standard normal distribution curve), and the estimated proportion of perceived self‐efficacy in the prevention of NCDs of 50% (0.5), due to the lack of prior research. *n* = (1.96)^2^ 0.50(1 − 0.50)/(0.05)^2^, *n* = 384. Considering a design effect of 1.5, the initial sample size was 576. After accounting for a 10% nonresponse rate, the final sample size increased to 634.

The participants in the study were selected using a multistage sampling technique. In the first stage, districts (woredas) were selected using probability proportional to size, which included three kebeles from the administrative town and three from each selected woreda. In the second stage, kebeles from each woreda were selected using simple random sampling: Nine kebeles were selected using simple random sampling: three from the administrative town and six from the selected woredas.

In the third stage, households were selected using systematic random sampling, and the sampling frame was developed using the most recent administrative household registry obtained from local authorities. Household selection was performed using a calculated sampling interval (k = total households/sample size). Within selected households, eligible participants were identified, and if more than one eligible adult was present, one was selected using a lottery method. If a household did not have any eligible participants, the next household was considered. Additionally, if eligible respondents were unavailable for data collection, at least two visits were conducted to attempt to reach them.

### 2.4. Study Variables

The dependent variable was perceived self‐efficacy in preventing NCDs, categorized as high or low.

The independent variables were included: sociodemographic and economic factors (sex, age, occupational status, marital status, educational status, family size, and household wealth index), behavioral and health‐related variables (known family history of NCDs, health insurance coverage, perceived general health status, and knowledge about the risk factors of NCDs), and HBM constructs (risk perception, perceived benefit, perceived barrier, and perceived cues to action). We have explicitly defined confounders as variables associated with both exposure and outcome but not on the causal pathway. These include age, sex, educational status, income, and marital status. These were controlled in the multivariable logistic regression model.

### 2.5. Definition of Confounding Variables

In this study, confounding variables were defined as variables that are associated with both the exposure and the outcome but are not considered to lie on the causal pathway between them. Based on theoretical considerations and prior empirical evidence, age, sex, educational status, household wealth index (income proxy), and marital status were identified as potential confounders. These variables were retained in the multivariable logistic regression model irrespective of their statistical significance to control for confounding effects.

### 2.6. Application of the HBM

This study was conceptually guided by the HBM, which posits that health behavior is influenced by perceived susceptibility, perceived severity, perceived benefits, perceived barriers, cues to action, and self‐efficacy. In this study, HBM constructs (risk perception, perceived benefits, perceived barriers, and cues to action) were operationalized as independent explanatory variables to examine their association with perceived self‐efficacy in preventing NCDs. The HBM constructs were not modeled as mediators due to the cross‐sectional design, which does not allow assessment of temporal ordering. Additionally, interaction terms were not included; therefore, effect modification was not formally tested. All HBM variables were treated as independent predictors in the multivariable logistic regression model while controlling for predefined confounders.

### 2.7. Measurement of Variables

#### 2.7.1. Self‐Efficacy

Perceived self‐efficacy was assessed using five items adapted from the HBM, designed to measure participants′ confidence in engaging in health‐promoting behaviors [17, 23]. Each item provided binary response options (yes/no). Responses indicating confidence were scored as 1 (“yes”), whereas those reflecting a lack of confidence were scored as 0 (“no”). The total score was obtained by summing the responses across all five items, yielding a composite self‐efficacy score ranging from 0 to 5, with higher scores indicating greater perceived self‐efficacy. For analysis, perceived self‐efficacy was categorized using a predefined cutoff point: Scores of 0–2 were classified as low perceived self‐efficacy, whereas scores of 3–5 were classified as high perceived self‐efficacy, consistent with previous studies [24]. The self‐efficacy scale showed satisfactory reliability in the study, with a Cronbach′s *α* of 0.74.

#### 2.7.2. Risk Perception of NCDs

Risk perception of NCDs was evaluated using 10 items derived from the HBM. The assessment consisted of five items addressing perceived susceptibility and five items addressing perceived severity. Each item featured a binary response option (yes = 1, no = 0). Scores for each item were summed to produce a composite score ranging from 0 to 10. This composite score was divided into two categories: low risk perception (scores between 0 and 5) and high‐risk perception (scores between 6 and 10). Higher scores reflect high perceived risk perception [25, 26].

#### 2.7.3. Perceived Benefits of Health Actions

The benefits perceived from health‐related actions were evaluated using seven yes/no questions based on the HBM. Each question assessed whether participants believed that specific health behaviors could lower their risk of developing NCDs. A “yes” response was scored as 1, indicating agreement with the perceived benefit, whereas a “no” response was scored as 0, indicating disagreement. These scores were totaled to create a composite perceived benefit score ranging from 0 to 7, with higher scores representing a stronger belief in the advantages of preventive health behaviors. For analysis, the scores were categorized into two groups: low perceived benefit (0–3) and high perceived benefit [4–7] [23].

#### 2.7.4. Perceived Barrier of Health Actions

Perceived barriers to health actions were evaluated through seven yes/no questions, which examined participants′ obstacles to adopting preventive behaviors NCDs, such as maintaining a healthy diet, engaging in physical activity, and attending regular health checkups. Responses were coded as 1 (“yes”) if the participant reported experiencing the barrier and 0 (“no”) if they did not. The total barrier score ranged from 0 to 7, with higher scores indicating greater perceived barriers. For analysis purposes, perceived barriers were classified into two categories: low perceived barriers (scores of 0–3) and high perceived barriers (scores of 4–7) [23].

#### 2.7.5. Cues to Action

Cues to action were measured using six binary (yes/no) questions that evaluated exposure to triggers or motivations encouraging individuals to engage in preventive health behaviors. These triggers included sources such as health education, media messages, advice from healthcare providers, and experiences of illness within family members. The item with the highest proportion of affirmative responses was coded as 1 (“yes”), whereas negative responses were coded as 0 (“no”). The total score could range from 0 to 6. Participants were categorized into two groups: those with low cues to action (scores of 0–3) and those with high cues to action (scores of 4–6) [27].

#### 2.7.6. Age of the Participants

Participants are divided into three distinct age groups: young adults (18–35 years), middle‐aged adults (36–50 years), and older adults (51 years and above) [23].

#### 2.7.7. Knowledge About NCD Risk Factors

We used 11 “yes” or “no” questions to assess participants′ knowledge of NCD risk factors. Participants received a score ranging from 0 to 11 based on their responses. Those who scored 70% or higher on the knowledge questions were classified as having “good knowledge,” whereas those who scored below 70% were categorized as having “unsatisfactory (poor) knowledge.” This classification is based on previously published research [28, 29].

#### 2.7.8. Participants Perceived General Health Status

A 5‐point Likert scale was used to evaluate participants′ perceived general health status, with options ranging from “very good” to “very poor.” For this study, health status was categorized into three groups: “good” or “very good” to indicate good health; “poor” or “very poor” to indicate poor health; and “medium” to represent medium health.

#### 2.7.9. Household Wealth Index

The study applied principal component analysis (PCA) to simplify large datasets and evaluate household socioeconomic status. A range of domestic assets was used as variables in the PCA, resulting in the creation of wealth quantiles [30]. For this research, the household wealth index was divided into three categories: poor, middle, and rich.

### 2.8. Data Collection Techniques, Procedures, and Quality Control

A structured questionnaire was developed for the interview by adapting insights from previous research on perceived self‐efficacy and related factors concerning NCDs [23–25, 27–30]. Data collection was conducted using the KoboToolbox a mobile application. The questionnaire included sections on participants′ sociodemographic and economic status, known family history of NCDs, health insurance coverage, perceived overall health status, perceived self‐efficacy in preventing NCDs, knowledge of NCDs risk, risk perception of NCDs, perceived barrier, perceived benefit, and cues to action of health‐related behavior [22]. Data were collected by four trained data collectors and two supervisors, all of whom were proficient in the local language and had backgrounds in health sciences. Before the data collection commenced, participants were informed of the study′s objectives, potential risks and benefits, confidentiality measures, and their right to decline participation or withdraw from the study at any time. Verbal informed consent was obtained from all participants. Interviews took place in the participants′ homes, with data collectors visiting each household in the selected kebeles until the required sample size was achieved. The questionnaire was initially created in English, then translated into the local language, and subsequently translated back into English to ensure consistency. Data collectors received 2 days of training before the actual data collection began. A pretest was conducted with 5% of the sample size in kebeles outside the study area, leading to necessary modifications in the questionnaire. Throughout the data collection process, data collectors were observed, and the collected data were checked for consistency.

### 2.9. Statistical Data Analysis and Processing

A KoboToolbox was used to collect the data, which was then exported to an Excel spreadsheet, examined for consistency, missing values, and completeness. The data were coded and then transferred to STATA Version 16 for analysis. For continuous data, means, standard deviations (SDs), or medians (interquartile ranges [IQRs]) were calculated, whereas frequencies and percentages were employed to characterize categorical data. Perceived self‐efficacy was treated as a binary outcome variable (high vs. low).

Bivariate logistic regression analysis was first performed to examine the association between each independent variable and perceived self‐efficacy. Variables with *p* values < 0.25 were considered for inclusion in the multivariable model as a screening criterion to avoid premature exclusion of potentially important predictors, consistent with purposeful model‐building approaches. In addition, variables were selected based on theoretical relevance, prior empirical evidence, and their potential role as confounders. Age, sex, educational status, income, and marital status were retained as a priori confounders regardless of statistical significance. The final multivariable model demonstrated acceptable goodness‐of‐fit (Hosmer–Lemeshow test *p* = 0.234). No evidence of multicollinearity was observed (mean VIF = 3). Finally, adjusted odds ratio (AOR) with 95% confidence intervals (CIs) was computed, and statistical significance in the final model was determined at *p* < 0.05.

### 2.10. Ethical Considerations

The study received ethical approval under protocol unique number of duirb/044/23‐06 from the Institutional Review Board (IRB) of Dilla University, College of Health Sciences and Medicine. It was conducted as part of the research project titled “Community Risk Perception and Health Promotion Behavior Towards Common NCDs Among Adult Residents of the Gedeo Zone, Southern Ethiopia.” The ethical protocol number aligns with that of a previously published paper [22], as both studies are components of the same project funded by Dilla University. This approval letter was presented to the appropriate administrative offices and other relevant stakeholders to obtain official authorization for the research. Prior to participation, individuals were provided with a brief explanation of the study′s importance and asked to give either verbal or written informed consent. Participants were assured of their right to accept or decline consent freely. Confidentiality and anonymity of the collected data were maintained throughout all stages of the research. All procedures adhered strictly to applicable guidelines and regulations.

## 3. Results

### 3.1. Sociodemographic and Economic Characteristics of Respondent

Out of the 634 samples used in this study, 620 individuals participated, yielding a 97.8% response rate. Of these participants, three‐fifths (61%) were women. The mean age of the study participants was 38.1 (±12.8) years, and above one‐third (35.1%) of the participants were found in the middle‐aged group. The mean family size was 4.4 ± 1.7, with above two‐thirds of the households having more than four family members. Above one‐third of the participants (36.3%) had completed secondary education, and nearly one‐third of the total participants (32.3%) were housewives in occupational status. Out of the participants, more than one‐third (38.7%) did not have health insurance, and 268 (59.4%) did not have a family history of NCDs.

Furthermore, based on the wealth index, nearly one‐third (32.9%) of the participants were classified as belonging to rich households. A total of 444 (71.6%) individuals had a good perceived general health status (Table 1).

**Table 1:** Sociodemographic and economic characteristics of residents in Gedeo Zone, Southern Ethiopia, 2023 (*n* = 620).

Variables	Categories	*N* (%)
Gender	Female	378 (61)
	Male	242 (39.0)
Age group	18–35 (young adults)	287 (46.3)
	36–50 (middle‐aged adults)	218 (35.2)
	≥ 51 (older aged adults)	115 (18.5)
Family size	≤ 4	195 (31.5)
	> 4	425 (68.5)
Educational status	No formal education	41 (6.6)
	Grades 1–8 (primary)	84 (13.6)
	Grades 9–12 (secondary)	225 (36.3)
	College and above	270 (43.5)
Occupational status	Student	80 (12.9)
	Government employed	185 (29.8)
	Housewife	200 (32.3)
	Merchant	110 (17.7)
	Others∗	45 (7.3)
Households′ wealth index	Poor	192 (31.5)
	Middle	221 (35.6)
	Rich	204 (32.9)
Marital status	Single	135 (21.8)
	Married	438 (70.6)
	Divorced	47 (7.6)
Known family History of NCDs	No	268 (59.4)
	Yes	253 (40.6)
Availability of health insurance	No	240 (38.7)
	Yes	380 (61.3)
Participants perceived general health status	Poor	74 (112.0)
	Moderate	102 (16.4)
	Good	444 (71.6)

*Note:* Asterisk “∗” denotes farmer, daily laborer, and pension.

### 3.2. Knowledge About NCD Risk Factors

Nearly 7 in 10 participants (69.2%) had heard about NCD risk factors. Above three‐fourths (76.8%) of the participants know that not exercising is a risk factor for NCDs. A total of 397 (64.0%) are aware that getting older raises the risk of NCDs. Moreover, the majority (81.1%) of the participants know that cigarette addiction (such as chewing or smoking) increases the risk of NCDs, and two‐thirds (66.3%) of the participants know that overweight/obesity is a risk factor for NCDs.

Finally, 474 (76.5%, 95% CI: 73.0, 79.6%) of the 620 participants had good knowledge of the risk factors for NCDs, and 146 (23.5%) of the participants had poor knowledge of the risk factors for NCDs (Table 2).

**Table 2:** Knowledge of the noncommunicable disease risk factors among residents of the Gedeo Zone, Southern Ethiopia, 2023 (*n* = 620).

Knowledge of NCD risk factors items	Response	
	Yes (%)	No (%)
Have you ever heard about NCDs risk factors?	429 (69.2)	191 (30.8)
Does avoiding unhealthy dietary habits help to prevent common NCD?	418 (67.4)	202 (32.6)
Are NCDs at risk because of family history?	450 (72.6)	170 (27.4)
Is a lack of exercise a risk factor for NCDs?	476 (76.8)	144 (23.2)
Does aging or getting older increase the risk of NCD?	397 (64.0)	223 (36.0)
Do environmental variables like air pollution and water pollution increase the incidence of NCDs?	417 (67.3)	203 (32.7)
Is drinking alcohol a risk factor for NCDs?	472 (76.1)	148 (23.9)
Does cigarette addiction (such as chewing or smoking) increase the risk of NCDs?	503 (81.1)	117 (18.9)
Is high blood pressure is a risk factor for NCDs?	415 (66.9)	305 (33.1)
Is high blood glucose is a risk factor for NCDs?	379 (61.1)	241 (38.9)
Does overweight/obesity is a risk factor for NCDs?	411 (66.3)	209 (33.7)

### 3.3. Risk Perception and Perceived Health Action for NCDs

In this study, the HBM constructs were used to assess participants′ risk perception and preventive health actions regarding NCDs. Individual risk perception reflects both the perceived severity of the illness and susceptibility to contracting it; accordingly, 343 (55.3%) participants reported a high‐risk perception of NCDs. Regarding preventive health actions, more than three‐fourths (77.9%) of participants had a high perceived benefit of engaging in health‐promoting behaviors, whereas 367 (59.2%) participants reported a lower perceived barrier to these actions. Additionally, 339 (54.7%) participants had higher cues to action to adopt health‐promoting behavior (Table 3).

**Table 3:** Distribution of health belief model constructs among study participants (*N* = 620).

Variables	Category	Frequency (*n*)	Percentage (%)
Risk perception of NCDs	Low	277	44.7
	High	343	55.3
Perceived benefit	Low	150	24.2
	High	470	75.8
Perceived barrier	Low	367	59.2
	High	253	40.8
Cues to action	Low	281	45.3
	High	339	54.7

### 3.4. Perceived Self‐efficacy in the Prevention of NCDs

**Figure 1:**
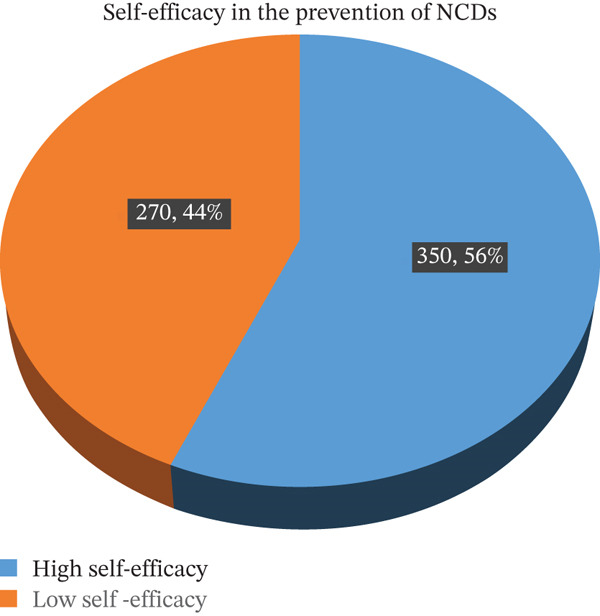
Self‐efficacy on the prevention of noncommunicable diseases among residents of the Gedeo Zone, Southern Ethiopia, 2023.

In terms of perceived self‐efficacy, the item with the highest proportion was “I have information about NCD prevention,” which had a proportion of 413 (66.6%). Conversely, the item with the lowest proportion was “I am confident in performing actions to maintain my health,” with a proportion of 266 (42.9) (Table 4). Overall, 350 (56.5%; 95% CI: 52.5–60.3) participants had high self‐efficacy toward engaging in NCD preventive behaviors (Figure 1).

**Table 4:** Self‐efficacy related to NCD prevention behaviors among participants (*N* = 620).

Self‐efficacy items	Yes *n* (%)	No *n* (%)
I am confident in performing actions to maintain my health.	266 (42.9)	354 (57.1)
I believe I could make changes to reduce NCD risk.	386 (62.3)	234 (37.7)
I have participated in health screening for NCDs.	319 (51.5)	301 (48.5)
I have information about NCD prevention.	413 (66.6)	207 (33.4)
I believe there is something I can do to lower my NCD risk.	385 (62.1)	235 (37.9)

### 3.5. Factors Associated With Perceived Self‐Efficacy in the Prevention of NCDs

The bivariate logistic regression results revealed that a good perceived self‐efficacy in NCD prevention was significantly related to the male gender, older adults, secondary, and college and above level education, other in occupational status, health insurance, good perceived general status, knowledge of NCD risk factors, risk perceptions of NCDs, perceived benefit, perceived barrier, and perceived cues to action at a *p* value of less than 0.25.

The multivariable logistic regression model revealed that the following factors were significantly associated (a *p* value of less than or equal to 0.05) with perceived self‐efficacy in the prevention of NCDs: middle‐aged and older adults, secondary in educational status, good perceived general health status, perceived benefit of health action, perceived barrier of health behaviors, and perceived cues to action (Table 5). To reduce potential bias from improper adjustments, the multivariable model incorporated theoretically relevant variables and predefined confounders. The AORs presented reflect associations after accounting for these factors and should not be interpreted as causal relationships.

**Table 5:** Multivariable logistic regression analysis of factors associated with high perceived self‐efficacy in preventing NCDs among adult residents of Gedeo Zone, Southern Ethiopia, 2023 (*n* = 620).

Variable	Category	High *n* (%)	Low *n* (%)	Crude OR (95% CI)	Adjusted OR (95% CI)	*p*
Age group	18–35 years	155 (44.3)	132 (48.9)	1.00	1.00	
	36–50 years	119 (34.0)	99 (36.7)	1.02 (0.71–1.46)	1.81 (1.09–3.01)	0.021
	≥ 51 years	76 (21.7)	39 (14.4)	1.66 (1.06–2.60)	4.31 (2.08–8.07)	< 0.001
Educational status	No formal education	12 (3.4)	29 (10.7)	1.00	1.00	
	Grades 9–12	140 (40.0)	85 (31.5)	3.98 (1.93–8.22)	2.86 (1.13–7.26)	0.026
Participants perceived general health status	Poor	22 (6.3)	52 (19.3)	1.00	1.00	
	Good	295 (84.3)	149 (55.2)	4.68 (2.74–7.99)	2.94 (1.38–6.28)	0.005
Perceived benefit	Low	31 (8.9)	119 (39.3)	1.00	1.00	
	High	319 (91.1)	151 (60.7)	8.11 (5.22–12.59)	5.54 (3.32–9.25)	< 0.001
Perceived barrier	Low	220 (62.9)	147 (54.4)	1.00	1.00	
	High	130 (37.1)	123 (45.6)	0.71 (0.51–0.98)	0.58 (0.38–0.88)	0.011
Cues to action	Low	95 (27.1)	186 (68.9)	1.00	1.00	
	High	255 (72.9)	84 (31.1)	5.94 (4.19–8.43)	4.35 (2.75–6.88)	< 0.001

The odds of having high perceived self‐efficacy were significantly higher among middle‐aged adults compared with young adults (AOR = 1.81; 95% CI: 1.09–3.01). Similarly, older adults had 4.31 times higher odds of having high perceived self‐efficacy compared with young adults (AOR = 4.31; 95% CI: 2.08–8.07). Participants with secondary education had higher odds of high perceived self‐efficacy compared with those without formal education (AOR = 2.86; 95% CI: 1.13–7.26).

Individuals who perceived their general health status as good had increased odds of high self‐efficacy compared with those who perceived their health as poor (AOR = 2.94; 95% CI: 1.38–6.28). Participants with high perceived benefits of health actions had higher odds of high perceived self‐efficacy compared with those with low perceived benefits (AOR = 5.54; 95% CI: 3.32–9.25).

Conversely, participants with high perceived barriers had 42% lower odds of high perceived self‐efficacy compared with those with low perceived barriers (AOR = 0.58; 95% CI: 0.38–0.88). Moreover, participants with high perceived cues to action had 4.35 times higher odds of high perceived self‐efficacy compared with those with low cues to action (AOR = 4.35; 95% CI: 2.75–6.88) (Table 5).

## 4. Discussion

Self‐efficacy is an essential aspect of motivation and behavioral change [31], and a person is more likely to carry out a behavior if they have a strong belief in their capacity to accomplish it [32]. The objective of this study was to assess the perceived self‐efficacy and associated factors in preventing NCDs among adult residents of the Gedeo Zone, Southern Ethiopia. This study used data collected as part of a broader research project titled “Community Risk Perception and Health Promotion Behavior Towards Common NCDs Among Adult Residents of the Gedeo Zone.” Although a previous publication from this project examined health‐promoting behaviors, the present analysis uses a separate dataset and focuses specifically on perceived self‐efficacy and its determinants [22]. This study found that 56.5% (95% CI: 52.5%–60.3%) of participants had high perceived self‐efficacy in preventing NCDs. This finding was consistent with a study conducted in Cambodia, which revealed that 58.1% of the participants perceived self‐efficacy in NCD prevention [33], and an Indonesian study that differentiated positive and negative self‐efficacy found that 53.7% of participants showed positive self‐efficacy [34]. In addition, this result was in line with a prior Ethiopian study that found 52.5% of participants had high perceived self‐efficacy; however, the latter study was aimed at determining perceived self‐efficacy in patients with diabetes at public hospitals [24].

This result, however, was lower than that of a study carried out in Indonesia that evaluated the degree of community self‐efficacy of coronary heart disease and found that 62.86% of the respondents had high self‐efficacy for coronary heart disease [35]. The primary distinction between the two studies was the previous study focused on self‐efficacy in preventing a single NCD (coronary heart disease), and our study focused on self‐efficacy in preventing NCDs. Furthermore, the current study′s sample size was higher than that of the previous study. Additionally, the results were lower than those of a Chinese study that reported the percent score index and found that 78.1% of participants showed self‐efficacy in NCD‐preventing activity [32]. The possible explanations for this disparity include variations in study settings, variable measurement, sampling techniques, sample size, and social, economic, and demographic factors.

The study revealed that the participants′ age had a significant relationship with perceived self‐efficacy of preventing NCDs, with middle‐aged and older aged adults having higher perceived self‐efficacy of preventing NCDs than young adults. This result was consistent with a South African study that showed variations in perceived self‐efficacy by age group [23]. The possible explanations for this finding could be that people are more aware of health risks and the value of preventative measures as they age. In addition, as the age increases, the chance of exposure to NCD is more likely to increase; this can be a potential motivator to take preventive action. Thus, awareness and exposure to NCD might boost their confidence in taking charge of their health and changing their healthy habits.

According to this study, a high perceived level of self‐efficacy in preventing NCDS was linked to secondary educational status. The link between perceived self‐efficacy and secondary education emphasizes how education helps people understand health information related to NCDs and make wise decisions. Better health literacy is frequently associated with educational achievement and can boost confidence in taking preventative measures against NCDs [36].

According to the current study, participants with good perceived health status had high perceived self‐efficacy in preventing NCDs. This result was consistent with Iranian studies that examined the association between self‐efficacy and overall health status [37, 38]. The finding might be due to high perceived general health status motivating individuals to adopt healthier behaviors. This motivation increases continued engagement in healthy behavior and enhances self‐efficacy in preventing NCDs. In addition, high perceived health status enhances coping mechanisms and self‐efficacy in lifestyle decisions and promotes self‐efficacy about NCD prevention.

This study indicates that those perceiving high barriers to health actions could experience lower self‐efficacy in preventing NCDs. This association was explained by the HBM, where an individual believes health barriers and benefits of taking action play a significant role in influencing health behaviors; individuals who perceive high barriers to healthy actions (such as cost, accessibility, and availability) might not be motivated to overcome these barriers, which lowers their perception of self‐efficacy and may result in decreasing the probability of an individual taking preventive health behavior [39]. According to Bandura′s social cognitive theory (SCT), one of the main factors influencing behavior change is self‐efficacy [40]. When people see significant barriers to taking health‐related actions, they may question their capacity to overcome them, which lowers their self‐efficacy and deters them from taking preventive action [41]. In this study, self‐efficacy in preventing NCDs had a positive relationship with the perceived benefit of health action. The possible explanation for the findings is that behavior change is initiated by the expectation that certain health‐related acts will result in favorable outcomes, and self‐efficacy increases when people understand the benefit of taking preventive measures (such as exercising or eating a healthy diet), making them more likely to engage in health‐promoting behaviors [42].

This study found a significant association between higher self‐efficacy in preventing NCDs and high perceived cues to action. The HBM explained that individuals are more likely to adopt health‐promoting behaviors when they perceive a cue to action (i.e., reminders [symptoms], advice from healthcare providers, or health campaigns) that prompt them to take action, and individuals who receive strong cues to action are empowered and feel more capable of making health‐related choices, which enhances their perceived self‐efficacy against NCDs. According to SCT, social environments such as family or community health initiatives provide higher perceived cues to action that can act as model behaviors. People′s self‐efficacy, or belief in their ability to carry out health‐promoting behaviors, rises when they observe others successfully carrying out these behaviors [41, 43].

Since cross‐sectional design cannot prove causality, it is not possible to establish temporal relationships between the predictors and perceived self‐efficacy. Therefore, the observed associations should not be interpreted as causal effects. Although multivariable adjustments were made to account for confounding factors, the possibility of residual confounding cannot be ruled out. Future studies should utilize longitudinal or intervention‐based designs to examine changes in perceived self‐efficacy over time and to assess the impact of specific public health interventions targeting NCD prevention. Although the study was conducted in the Gedeo Zone, the findings may provide useful insights for similar rural and semiurban populations in Ethiopia. However, variations in socioeconomic conditions, health service access, and cultural practices may influence perceived self‐efficacy in other regions; therefore, caution should be exercised when generalizing the results nationally.

### 4.1. Strength and Limitation of the Study

This study has several strengths. It employed a community‐based design with a relatively large sample size, which enhances the representativeness and precision of the findings. Additionally, the use of the HBM provided a strong theoretical framework for assessing behavioral factors, and multivariable logistic regression analysis was conducted to control for potential confounders. This study also presents several limitations. The cross‐sectional nature of the study limits the ability to establish causal relationships between predictors and perceived self‐efficacy. The exclusion of individuals with previously diagnosed NCDs may limit the generalizability of the findings to populations already living with these conditions. Thirdly, the use of self‐reported responses may introduce social desirability bias. Finally, the use of binary response options for measuring self‐efficacy may have reduced the variability of responses compared with multicategory Likert scales, which could result in some loss of information.

### 4.2. Public Health Implications

The findings carry significant implications for NCD prevention initiatives in Ethiopia. Elements linked to perceived self‐efficacy, such as encouraging a healthy diet, promoting physical activity, and enhancing health education, align with the WHO “Best Buys” for NCD prevention. These recommendations prioritize cost‐effective, population‐level strategies, including tobacco control, salt intake reduction, and public awareness campaigns [44]. Several of the recommended behavioral interventions identified in this study align with the WHO “Best Buy” interventions for NCD prevention, including tobacco control policies, promotion of physical activity, and reduction of unhealthy diets. These cost‐effective strategies are considered among the most feasible approaches for reducing the burden of NCDs in low‐ and middle‐income countries [45].

This study did not examine policy exposure, leaving it uncertain whether the observed self‐efficacy levels can be attributed to current national initiatives. Future longitudinal or quasiexperimental research is necessary to assess policy impacts and integrate self‐efficacy measures into monitoring and evaluation frameworks. The findings are primarily applicable to adults in comparable sociodemographic and healthcare system settings. However, caution should be exercised when applying these results to rural or densely urbanized populations in Ethiopia, as contextual factors and service accessibility may vary.

Ethiopia has recognized the growing burden of NCDs and developed national strategies aligned with the WHO Global Action Plan for NCD prevention and control. These strategies emphasize community‐based prevention, health promotion, and early detection through the primary healthcare system. However, evidence regarding the behavioral and psychosocial determinants of preventive practices remains limited. Understanding perceived self‐efficacy among communities can therefore help inform national NCD prevention programs and guide the development of effective behavior‐change interventions.

### 4.3. Conclusion and Recommendations

More than half of the study participants demonstrated high perceived self‐efficacy toward NCD prevention. Age, education, perceived health status, perceived benefits, perceived barriers, and cues to action were significantly associated with self‐efficacy. Interventions aimed at strengthening health education, reducing perceived barriers, and increasing community‐level cues to action may improve individuals′ confidence in engaging in NCD preventive behaviors.

Strengthen the engagement of community leaders and local organizations to promote awareness campaigns within the community concerning the perceived benefits of health actions. Promote policies designed to advocate public health initiatives focused on NCD prevention. Strategies may entail partnerships with the government for health education to be instituted in the school curricula and community programs. Through community assessments, barriers to health actions perceived by the community should be identified and addressed; solutions may include access to healthcare facilities and affordable health services. Moreover, community‐based interventions should be encouraged to enable people to develop their self‐efficacy in preventing NCDs through seminars or support groups, which lead to better health promotion practices and, ultimately, to collective action for NCD prevention.

**Nomenclature**
AOR: adjusted odds ratio
COR: crude odds ratio
HBM: health belief model
IRB: Institutional Review Board
IQR: interquartile range
NCDs: noncommunicable disease
PCA: principal component analysis
SCT: social cognitive theory
SD: standard deviation
VIF: variance inflation factor

## Author Contributions

H.E.H. and B.G.D. conceptualized the original draft, investigation, methodology/statistical analysis, tool development, and writing of the paper. M.A. and Y.A.W. participated in the investigation, software validation, statistical analysis, data curation, and writing of the paper.

## Funding

This study was financially supported by Dilla University. However, the funder had no role in the design, data extraction, analysis, and interpretation of the data, in writing the manuscript, or in the decision to submit it for publication.

## Disclosure

All authors reviewed and approved the final manuscript and agreed to be accountable for all aspects of the work.

## Ethics Statement

An ethical approval letter was obtained from the Institutional Review Board (IRB) of Dilla University, College of Health Sciences and Medicine. Each participant gave verbal informed consent after being briefed on the significance of the study. All methods were performed in accordance with relevant guidelines and regulations.

## Consent

The authors have nothing to report.

## Conflicts of Interest

The authors declare no conflicts of interest.

## Data Availability

Data are available on request from the authors.
