# Correction to “Perceived Self‐Efficacy and Its Determinants for Noncommunicable Disease Prevention Among Adults in Southern Ethiopia: A Community‐Based Cross‐Sectional Study”

**DOI:** 10.1155/bmri/9896313

**Published:** 2026-07-27

H. E. Hareru, M. Abebe, B. G. Debela, T. T. Mamo, and Y. A. Wondimagegne, “Perceived Self‐Efficacy and Its Determinants for Noncommunicable Disease Prevention Among Adults in Southern Ethiopia: A Community‐Based Cross‐Sectional Study,” *BioMed Research International* 2026 (2026): 3904968, https://doi.org/10.1155/bmri/3904968.

In the abovementioned article, there was an error in the Author Contributions section, where the contributions of co‐author Tizalegn Tesfaye Mamo (T.T.M.) were inadvertently omitted. The corrected section appears below:

## Author Contributions

H.E.H. and B.G.D. conceptualized the study, prepared the original draft, conducted the investigation, developed the methodology, performed the statistical analysis, developed the study tools, and wrote the manuscript. M.A., T.T.M., and Y.A.W. participated in the investigation, software validation, statistical analysis, data curation, and manuscript writing.

We apologize for this error.

